# Pseudohyponatremia: Mechanism, Diagnosis, Clinical Associations and Management

**DOI:** 10.3390/jcm12124076

**Published:** 2023-06-15

**Authors:** Fahad Aziz, Ramin Sam, Susie Q. Lew, Larry Massie, Madhukar Misra, Maria-Eleni Roumelioti, Christos P. Argyropoulos, Todd S. Ing, Antonios H. Tzamaloukas

**Affiliations:** 1 Department of Medicine, Division of Nephrology, University of Wisconsin School of Medicine and Public Health, Madison, WI 53705, USA; faziz@wisc.edu; 2 Department of Medicine, Zuckerberg San Francisco General Hospital, School of Medicine, University of California in San Francisco, San Francisco, CA 94110, USA; ramin.sam@ucsf.edu; 3 Department of Medicine, School of Medicine and Health Sciences, George Washington University, Washington, DC 20052, USA; sqlew@gwu.edu; 4 Department of Pathology, Raymond G. Murphy Veterans Affairs Medical Center, University of New Mexico School of Medicine, Albuquerque, NM 87108, USA; larry.massie@va.gov; 5 Department of Medicine, Division of Nephrology, University of Missouri, Columbia, MO 65211, USA; misram@health.missouri.edu; 6 Department of Medicine, Division of Nephrology, University of New Mexico School of Medicine, Albuquerque, NM 87106, USA; mroumelioti@salud.unm.edu; 7 Department of Medicine, Stritch School of Medicine, Loyola University Chicago, Maywood, IL 60153, USA; todd.ing@gmail.com; 8 Research Service, Department of Medicine, Raymond G. Murphy Veterans Affairs Medical Center, University of New Mexico School of Medicine, Albuquerque, NM 87108, USA; antonios.tzamaloukas@va.gov

**Keywords:** hyponatremia, pseudohyponatremia, pseudonormonatremia, pseudohypernatremia, serum sodium concentration, serum water sodium concentration, serum solids content, serum proteins, serum lipids, electrolyte exclusion effect, dilution effect, hyperviscosity

## Abstract

Pseudohyponatremia remains a problem for clinical laboratories. In this study, we analyzed the mechanisms, diagnosis, clinical consequences, and conditions associated with pseudohyponatremia, and future developments for its elimination. The two methods involved assess the serum sodium concentration ([Na]_S_) using sodium ion-specific electrodes: (a) a direct ion-specific electrode (ISE), and (b) an indirect ISE. A direct ISE does not require dilution of a sample prior to its measurement, whereas an indirect ISE needs pre-measurement sample dilution. [Na]_S_ measurements using an indirect ISE are influenced by abnormal concentrations of serum proteins or lipids. Pseudohyponatremia occurs when the [Na]_S_ is measured with an indirect ISE and the serum solid content concentrations are elevated, resulting in reciprocal depressions in serum water and [Na]_S_ values. Pseudonormonatremia or pseudohypernatremia are encountered in hypoproteinemic patients who have a decreased plasma solids content. Three mechanisms are responsible for pseudohyponatremia: (a) a reduction in the [Na]_S_ due to lower serum water and sodium concentrations, the electrolyte exclusion effect; (b) an increase in the measured sample’s water concentration post-dilution to a greater extent when compared to normal serum, lowering the [Na] in this sample; (c) when serum hyperviscosity reduces serum delivery to the device that apportions serum and diluent. Patients with pseudohyponatremia and a normal [Na]_S_ do not develop water movement across cell membranes and clinical manifestations of hypotonic hyponatremia. Pseudohyponatremia does not require treatment to address the [Na]_S_, making any inadvertent correction treatment potentially detrimental.

## 1. Introduction

Electrolyte measurements are the most frequently ordered blood tests in modern clinical chemistry laboratories [1]. The normal serum sodium concentration ([Na]_S_) varies from 137 to 142 mmol/L [2]. Hyponatremia represents the most common electrolyte disturbance seen in hospital practice [3]. Mild hyponatremia ([Na]_S_ between 130 and 134 mmol/L) occurs in 15–30% of hospitalized patients, and in 18% of nursing home individuals [4,5]. Multiple studies indicate significantly worse outcomes in patients with hyponatremia who are admitted for sundry reasons [6,7,8,9,10,11]. 

Serum volume consists of serum water and serum solids. Normally, the serum solids content (SSC) comprises 0.07 (or 7%) and the serum water content (SWC) comprises 0.93 (or 93%) of the volume of serum [12]. The SSC contains proteins and lipids, but no sodium. Sodium is carried exclusively in the SWC. Therefore, the sodium concentration in serum water ([Na]_SW_) is greater than the [Na]_S_. It is important to underline that the [Na]_SW_, not the [Na]_S_, determines the biological actions of sodium, including its effect as an osmotic agent in causing fluid shifts between the intracellular and extracellular compartments [13]. The relation between the [Na]_SW_ and the [Na]_S_ is expressed as [Na]_SW_ = [Na]_S_/SWC. 

Since sodium is present only in the SWC, true hyponatremia can best be defined as a “clinical condition in which the [Na]_SW_ is lower than normal”. Pseudohyponatremia, also known as spurious or artifactual hyponatremia, can be referred to as “a situation in which [Na]_SW_ is normal, but the reported [Na]_S_ is low”.

Pseudohyponatremia can cause therapeutic mishaps [14,15,16,17,18,19]. The early identification of pseudohyponatremia can prevent initiating measures that are directed towards correcting a falsely low [Na]_S_. This review aims to explain the underlying mechanisms, diagnosis and conditions associated with pseudohyponatremia, plus future developments aiming to eliminate it. 

## 2. Methods for Measuring Serum Sodium Concentration

In clinical laboratories, the sodium concentration is measured with flame emission spectrophotometry (FES) or ion specific electrodes (ISE). With the advent of FES in the 1940’s, [Na]_S_ measurement became an important laboratory function [20]. Currently, most clinical laboratories do not use FES. In the 1970s, with the introduction of ISE technology and the autoanalyzer-centric automation of various chemistry tests, [Na]_S_ measurement has become easier. 

The ISE methods can measure the sodium concentration via two approaches, directly and indirectly. The direct ISE method estimates the [Na]_S_ without requiring pre-dilution of the sample. In contrast, the indirect ISE method requires pre-measurement dilution of the sample for its estimation of the [Na]_S_. After their introduction in the 1970s, ISE methods have become the most popular approaches to measure the [Na]_S_ [2,21,22,23,24,25]. ISE methods were used to measure the [Na]_S_ by more than 99% of the laboratories that reported proficiency data for this measurement to the College of American Pathologists in 2015 [26,27].

An ISE apparatus measures the electrical potential across a sodium-selective membrane immersed in the to-be-tested sodium sample. The electrical potential depends on the sodium concentration in the sample. Both the direct and indirect ISE measure sodium electrical activity in serum water (the direct method in undiluted serum water and the indirect method in diluted serum water), not in undiluted or diluted serum, respectively [28,29]. The ISE devices are calibrated to express the activity as sodium concentration by comparing with aqueous solutions that mimic the ratio of SWC/serum. As a result of this accounting, the measuring devices report the [Na]_S_, not the measured [Na]_SW_. For both the direct and indirect ISE methods, the algorithms that convert the [Na]_SW_ to [Na]_S_ use a SWC/serum ratio of 0.93 for all samples measured, as this is the SWC ratio of the control solution used for calibration. The direct ISE method estimates the [Na]_S_ in serum or heparinized whole blood [30]. In the case of whole blood, this method avoids the need to separate plasma from cellular components using centrifugation. 

The indirect ISE method continues to be the most popular method for measuring the [Na]_S_. In 2006, more than two-thirds of clinical chemistry laboratories in the US used the indirect ISE method for the estimation of [Na]_S_ [27]. In 2011, Fortgens and Pillay found that most high-volume laboratories still used an indirect ISE method [1]. The authors of the present report sampled the chemistry laboratories of 14 medical school-affiliated hospitals and found that the auto-analyzers of 12 laboratories used the indirect ISE method. The indirect test is still more popular, because it has been incorporated into the panel of multiple routine tests carried out by an autoanalyzer. In the latter, a small sample of serum needs to be diluted into a larger volume, in order to enable many tests to be performed. 

## 3. Mechanisms of Pseudohyponatremia

The relation between the SSC and the SWC is traditionally expressed as SSC + SWC = 1, indicating that the SWC changes in the opposite direction and by the same magnitude when the SSC changes [31]. The actual SWC is slightly lower than the value of 1—SSC, because the SWC value includes the molecular volumes of crystalloids dissolved in serum water, in addition to the volume of water. These molecular volumes are too small to affect the accuracy of calculations involving the SWC, amounting to about 0.9% of the expression 1—SSC [12]. 

The proposed mechanisms of pseudohyponatremia when the [Na]_S_ is measured via the indirect ISE or FES, which require pre-measurement dilution of the serum specimen, include the following: (a) the electrolyte exclusion effect; (b) the dilution effect; and (c) the hyperviscosity effect. None of these three mechanisms operates when the [Na]_S_ is measured using the direct ISE. In addition, pseudohyponatremia has been reported as a result of mechanisms specific to certain medical conditions. These conditions are discussed in a later section of this report. 

### 3.1. Sodium Concentration Lowering by the Electrolyte Exclusion Effect

The electrolyte exclusion effect, also known as the volume displacement effect, can be defined as a decrease in the concentrations of electrolytes in whole serum because these electrolytes are contained only in the SWC [27,32]. Table 1 shows actual [Na]_S_ values at three different SWC values. The [Na]_SW_ is the same (151 mmol/L) in all three examples shown in this table. Any method that measures the sodium concentration in serum and not in serum water, e.g., FES, will underestimate the [Na]_SW_, and the degree of underestimation increases as the SSC increases and the SWC decreases. 

The indirect ISE method, which measures sodium concentration in the water fraction of a diluted serum sample, is subject to the electrolyte dilution effect, as will be shown in the next subsection. In contrast, the direct ISE approach, which measures sodium concentration in the water fraction of undiluted serum, is not affected by the electrolyte exclusion effect. The direct ISE values will be the same at all SWC values when the [Na]_SW_ is the same. For example, at a [Na]_SW_ of 151 mmol/L, the direct ISE will report a [Na]_S_ of 0.93 × 151 = 140.4 mmol/L at both an SWC = 0.93 and an SWC = 0.79. As shown in Table 1, the actual [Na]_S_ is 119.3 mmol/L at an SWC = 0.79. However, the [Na]_S_ reported with the direct ISE method eliminates pseudohyponatremia because it indirectly indicates the true value of the [Na]_SW_. 

Several studies have documented that the direct ISE method is not influenced by electrolyte exclusion. The [Na]_S_ measured by the direct ISE method was the same before and after removal of excess lipids from a hyperlipemic serum in one study [33]. In a second study, [Na]_S_ values measured with FES were substantially lower than the corresponding values measured by a direct ISE in hyperlipemic sera, while after removal of the lipids, the [Na]_S_ values measured via FES rose substantially and became almost identical to the values measured with a direct ISE [34]. In another study, progressively increasing the protein concentration in aqueous solutions had minimal effects on the concentrations of sodium and potassium measured by the direct ISE, but produced a progressive decrease in the concentrations of both cations measured with FES [23]. 

### 3.2. Sodium Concentration Lowering by the Dilution Effect

Dilution of a serum sample with high SSC prior to measurement of its [Na]_S_ combined with the electrolyte exclusion effect results in pseudohyponatremia [32]. The dilution factor for serum water (DFSW) is calculated as (volume of fluid added plus serum water volume)/(serum water volume) [32]. This factor increases progressively at progressively lower SWC values with use of the same volume of diluent [32]. 

Table 2 shows an example of the effect of dilution on the measurement of [Na]_S_ with an indirect ISE in a serum sample with normal SSC and two serum samples with high SSC values. The [Na]_SW_ was 151 mmol/L in all three samples. Note that the true values of the [Na]_S_ computed directly from the [Na]_SW_ and the SWC (Table 1) differed only slightly from the corresponding values of [Na]_S_ computed after measurement of the sodium concentration in the water of the diluted serum specimens when the [Na]_SW_ and the [Na]_S_ were computed assuming an SWC of 0.93 (Table 2). Therefore, the effect of dilution consists only in expressing the exclusion effect when the auto-analyzer algorithms for an indirect ISE compute the [Na]_S_ using an SWC of 0.93 and its corresponding DFSW. 

To recapitulate, in sera with the same [Na]_SW_ values, the electrolyte exclusion effect is responsible for the progressively lower [Na]_S_ values reported via an indirect ISE at progressively higher SSC values. Pre-measurement dilution allows expression of the electrolyte exclusion effect. 

### 3.3. Sodium Concentration Lowering by the Hyperviscosity Effect

The hyperviscosity effect becomes apparent when highly viscous serum specimens are diluted prior to measuring their sodium concentrations [35]. Pronounced hyperproteinemia, e.g., in multiple myeloma or Waldenstrom’s macroglobulinemia [36], causes serum hyperviscosity. When using pumps, e.g., roller ones, in apportioning serum and diluent to deliver the required volume of diluted serum to an automatic dilution device, hyperviscosity can cause a decrease in the delivered serum to the device that assays the sodium activity while the delivery of the non-viscous diluent is unimpeded, thus augmenting the electrolyte exclusion effect. A low temperature of a measured sample can increase this hyperviscosity effect [37]. This device-related decrease in serum sample delivery (hence, in sodium delivery) to a sodium analysis device causes pseudohyponatremia [38,39,40]. 

Overlack and coauthors reported that hyperviscosity accounted for the largest proportion of pseudohyponatremia cases in patients with multiple myeloma and hyperproteinemia [41]. Hyperviscosity contributed to pseudohyponatremia that was observed after immunoglobulin infusion [42], and in the hypercholesterolemic plasma of a patient with primary biliary cirrhosis [43]. In conclusion, the impaired delivery of serum with hyperviscosity to the sodium-measuring apparatus after pre-measurement dilution is purely a mechanical problem, and is unrelated to the electrolyte exclusion effect. Hyperviscosity does not influence the [Na]_S_ measured by placing a drop of serum on a microslide in an apparatus that uses a direct ISE [23]. 

## 4. Diagnosis of Pseudohyponatremia

One approach used to calculate the [Na]_SW_, and consequently to diagnose pseudohyponatremia, consists of dividing the [Na]_S_ reported by a method using pre-measurement dilution by the SWC [44]. Waugh developed the following empirical formula expressing SWC in 100 mL of serum [12]:

(1) $$100\;\times \;\mathrm{SWC}=99.1-0.73\times \left[SP\right]-1.03\times \left[SL\right]$$

where 99.1 is the volume of water contained in 100 mL of a crystalloid solution having the composition and concentrations of crystalloids in serum water; [SP] is the concentration of proteins in g/dL of serum; and [SL] is the concentration of lipids in g/dL of serum. Various other methods for estimating the SWC and [Na]_SW_ have been proposed [45,46,47,48,49,50,51,52]. SSC values lower than 0.07 may result in pseudonormonatremia in cases of hypotonic hyponatremia, or in pseudohypernatremia in cases of true normonatremia. Formula 1 suggests that a low plasma protein [PP] is the main cause of spurious hypernatremia or spurious normonatremia, since the normal values of plasma lipid [PL] are around 0.3 g/100 mL and the normal values of [PP] are around 8 g/100 mL. Several studies have confirmed this suggestion [50,51,53,54]. 

Musso and Bargman proposed that the first step in evaluating hyponatremia in asymptomatic patients on peritoneal dialysis consists of checking for pseudohyponatremia [55]. We suggest that pseudohyponatremia should be considered in all low [Na]_S_ values measured using an indirect ISE. Pseudohyponatremia is diagnosed directly in this case by measuring the [Na]_S_ with a direct ISE [56]. However, detecting whether a low [Na]_S_ value was caused by hypotonic hyponatremia, hypertonic hyponatremia, or pseudohyponatremia [57], and particularly whether there are combinations of pseudohyponatremia with other dysnatremic states when a low [Na]_S_ value is reported via the indirect ISE method, is based on measuring serum osmolality, and computing the osmol gap [58]. The osmol gap represents the difference between the measured serum osmolality and serum osmolarity, calculated as the sum 2 × [Na]_S_ + serum urea + serum glucose, where both the serum glucose and urea concentrations are in mmol/L [17,59]. 

**Figure 1:**
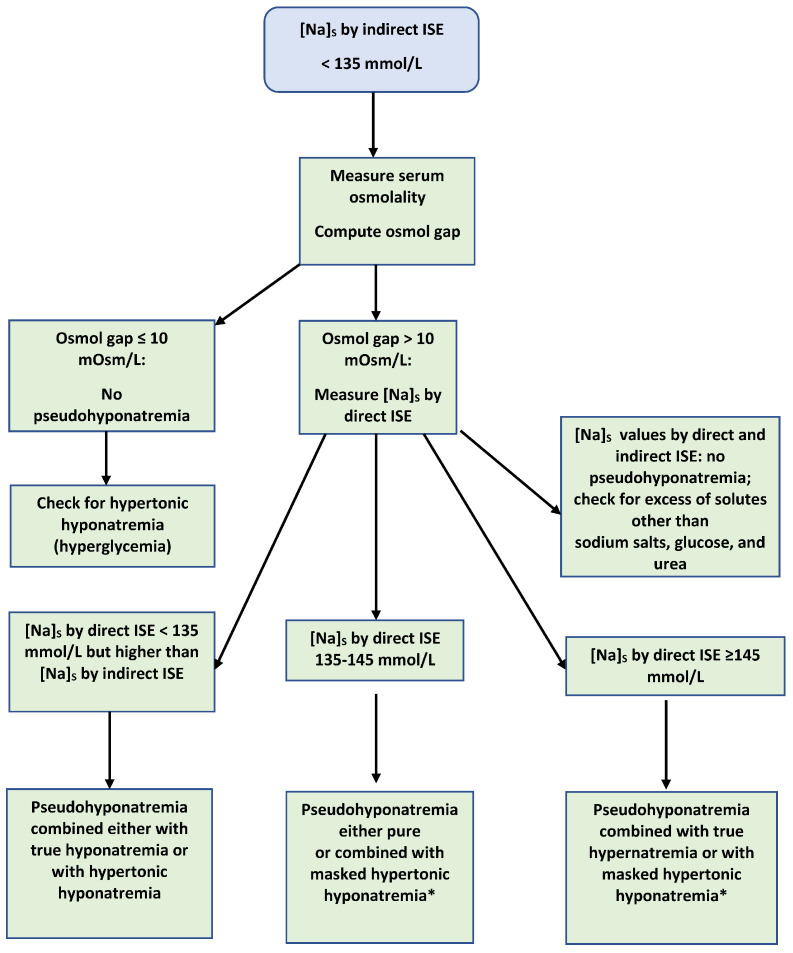
Diagnosis of pseudohyponatremia and accompanying dysnatremias. Osmol gaps that are calculated using the direct instead of the indirect [Na]_S_ value and are still enlarged indicate the presence in serum of a solute other than sodium salts, glucose, or urea. [Na]_S_ values < 135 mmol/L reported by direct ISE result from either hypotonic or hypertonic hyponatremia. Hyperglycemic states by far represent the most frequent cause of hypertonic hyponatremia. * Hypertonic hyponatremia masked by a mechanism causing hypernatremia, e.g., osmotic diuresis caused by hyperglycemia.

Figure 1 shows a “based on the osmol gap” scheme for the diagnosis of pseudohyponatremia and other dysnatremias potentially associated with it in cases of a low [Na]_S_ measured with the indirect ISE approach. Combinations of dysnatremias should be suspected in every case with an osmol gap that is larger than 10 mmol/L. Pseudohyponatremia is confirmed when the [Na]_S_ measured a direct ISE exceeds the corresponding indirect ISE value. In all instances of pseudohyponatremia, the osmol gap should be recalculated using the [Na]_S_ measured with a direct ISE. If the new osmol gap is within the normal range, pseudohyponatremia was the sole cause of the original gap. If the new osmol gap is less than the original, but still above the normal range, this means that pseudohyponatremia is combined with excesses of solutes other than sodium, glucose, or urea. Combinations of pseudohyponatremia with other dysnatremias that can be detected by large osmol gaps are encountered clinically. 

In addition to pseudohyponatremia, large osmol gaps are encountered in situations where there is a gain of solutes other than sodium, glucose, and urea in the serum [60]. Examples of endogenous solute gains include advanced chronic kidney disease [61] and the sick cell syndrome [62]. A large osmol gap from the gain in exogenous solutes distributed in total body water, e.g., ethanol [63], may be associated with both hypotonic hyponatremia and pseudohyponatremia (through hyperlipidemia). Gains in solutes with extracellular distribution cause hypertonic hyponatremia. A gain in exogenous extracellular solutes, e.g., mannitol [64], will raise the osmol gap. If such a solute is endogenous, i.e., glucose, [65] pseudohyponatremia (again through hyperlipidemia) with a large osmol gap may also be present.

## 5. Clinical Conditions Associated with Pseudohyponatremia

Table 3 lists clinical states in which pseudohyponatremia has been reported, including conditions associated with hyperproteinemia [41,66,67,68,69,70,71,72,73,74,75,76,77,78,79,80], hypertriglyceridemia [19,68,81,82,83,84,85,86,87,88,89,90,91,92,93,94,95,96,97] and hypercholesterolemia [98,99,100,101,102,103,104,105,106,107,108,109,110,111,112,113,114].

### 5.1. Hyperproteinemia

Hyperproteinemic diseases may produce multiple mechanisms for hyponatremia. In multiple myeloma, hyperproteinemia is the usual cause of pseudohyponatremia. Serum cholesterol levels are routinely low in patients with multiple myeloma because of increased low-density lipoprotein (LDL) clearance and the uptake of cholesterol by tumor cells [115]. However, pseudohyponatremia results from a combination of hyperproteinemia and hypercholesterolemia in patients with multiple myeloma who exhibit hypercholesterolemia [116]. Low [Na]_S_ values in multiple myeloma patients may represent combinations of pseudohyponatremia with other dysnatremias. Combinations of pseudohyponatremia and hypotonic hyponatremia are encountered when there are manifestations of myeloma that cause a relative excess of body water, e.g., the syndrome of inappropriate antidiuretic hormone secretion [117,118,119]. Hyponatremia is also encountered when paraproteins in sera have positive charges [120]. 

Monoclonal gammopathies may cause hyperproteinemia and hyperviscosity [121]. An infusion of immunoglobulins may cause pure pseudohyponatremia or a combination of pseudohyponatremia and hypertonic hyponatremia. Immunoglobulin preparations for intravenous infusion frequently contain 10% maltose solutions [122]. Maltose is metabolized by maltase contained in the brush border of renal proximal tubular cells [123]. In patients with renal dysfunction, the infusion of immunoglobulin solutions has been found to cause combinations of pseudohyponatremia due to hyperproteinemia and hypertonic hyponatremia that is secondary to maltose accumulation in the extracellular compartment [124,125]. Maltose present in the serum increases the osmol gap. Combinations of pseudohyponatremia and hypertonic hyponatremia have also been reported after infusions of sucrose-containing immunoglobulin preparations [126]. 

### 5.2. Hyperlipidemia

Severe hypertriglyceridemia may cause both pancreatitis and pseudohyponatremia [81,82,83,84]. The lipoprotein lipase, an enzyme of endothelial cells, catabolizes triglyceride-containing compounds including chylomicrons and very-low-density lipoprotein (VLDL). Asparaginase, a drug used for the treatment of hematologic malignancies and other malignant diseases, inhibits lipoprotein lipase activities [127]. The concentration of triglycerides in serum becomes elevated transiently after asparaginase administration [128]. In some instances, both serum cholesterol and serum triglyceride levels are elevated after asparaginase treatment [87]. 

### 5.3. Diabetic Ketoacidosis

As in immunoglobulin infusion, diabetic ketoacidosis (DKA) with elevated serum lipid levels may cause combined pseudohyponatremia and hypertonic hyponatremia. In addition, osmotic diuresis in combination with thirst and fluid intake may cause combinations of pseudohyponatremia, hypertonic hyponatremia and hypernatremia or hypotonic hyponatremia in hyperglycemic emergencies [65,129]. The presence and degree of dysnatremias masked by combined pseudohyponatremia and hypertonic hyponatremia can be detected by measuring the [Na]_S_ with a direct ISE and computing the [Na]_S_ that results from correcting the hyperglycemia [65]; monitoring the [Na]_S_ during treatment remains imperative [65]. 

Pseudohyponatremia in DKA may be encountered in the absence of an elevated SSC [96]. In this case, a low blood pH or other unknown conditions are thought to affect [Na]_S_ measurement with an indirect ISE [94]. The effect of very high glucose concentrations on the measurement of sodium concentration with ISE methods need further studies. In samples with extremely high glucose concentrations, one study reported finding spuriously high sodium concentrations when the [Na]_S_ was measured using a direct ISE, but not for an indirect ISE [130], while a second study reported spuriously high sodium concentrations measured with an indirect ISE, but not with a direct ISE [131].

### 5.4. Enzyme Mutations Causing Hypertriglyceridemia

Enzyme mutations, mainly of the lipoprotein lipase, may cause profound hypertriglyceridemia, and consequently pseudohyponatremia [132]. Several enzyme mutations causing hypertriglyceridemia have been reported [133,134,135,136,137,138,139].

### 5.5. Hypercholesterolemia Caused by Cholestasis

Liver diseases that are associated with cholestasis have been linked to pseudohyponatremia associated with hypercholesterolemia (Table 3). Cholesterol is transported in the blood by VLDL and lipoprotein X. The blood levels of lipoprotein X are elevated in cases of hypercholesterolemia, due to cholestasis [140,141]. Pseudohyponatremia that is secondary to severe hypercholesterolemia associated with use of certain drugs has also been reported [106,107]. Hepatitis with cholestasis has been observed as a complication of these medications, which include the antipsychotic quetiapine [142], trimethoprim-sulfamethoxazole [143], and the antiviral agent valacyclovir [144]. Alagille syndrome, an autosomal dominant disorder caused by mutations in genes JaG1 or NOTCH2 of the Notch signaling pathway, causes cholestasis and severe clinical manifestations from other organ systems [145].

### 5.6. Pseudohyponatremia in the Absence of Elevated Serum Solids Content

In addition to DKA, other conditions can cause pseudohyponatremia in the absence of an elevated SSC. Pseudohyponatremia associated with pseudohyperkalemia has been reported in heparinized plasma samples from patients with non-Hodgkin’s lymphoma [146] and acute lymphoblastic leukemia [147]. Some of the proposed mechanisms affecting the collected blood sample include the following: (a) lysis of white blood cells in heparinized blood samples with the release of potassium and ATP into the plasma, causing sodium influx into lymphocytes and pseudohyponatremia [147]; and (b) a defect in the cell membranes of red blood cells causing potassium to exit from red cells and sodium to enter these cells [148]. 

The combination of pseudohyperkalemia and pseudohyponatremia has also been observed in serum samples that were separated with some delay after blood sample collection in a patient with hereditary stomatocytosis; this is an autosomal dominant condition in which a defect in the red cell membrane leads to increased sodium influx into the red cells, which is counteracted in vivo by a large increase in sodium/potassium ATPase activity of the red cell membrane. After blood collection, the activity of the ATPase is diminished as a consequence of a decrease in the blood sample temperature and the reduced supply of ATP due to a decrease in glucose concentration of the serum sample, leading to the development of pseudohyperkalemia and pseudohyponatremia [149]. 

### 5.7. Differences in [Na]_S_ Values Measured by Different Direct ISE Apparatuses

When the degree of pseudohyponatremia is considered, differences between [Na]_S_ values measured with a direct ISE in a “point-of-care” (POC) setting in an intensive care unit using the blood gas apparatus and in the main hospital laboratory should be considered. The frequencies of discrepancies found in paired measurements between the two direct ISE apparatuses reported by Weld and co-investigators were 4.1% for a ≥4 mmol/L disagreement, 13.4% for a ≥3 mmol/L disagreement, and 36.2% for a ≥2 mmol/L disagreement; these authors identified the level of serum proteins as one source of disagreement, with measurements in the central laboratory being lower than the corresponding POC measurements at low serum protein levels, and higher than the POC measurements at high serum protein levels; the authors concluded that these disagreements were sufficient to affect conditions in which an accurate measurement of the [Na]_S_ is required, e.g., in the treatment of hyponatremia [150]. 

Other potential sources of discrepancies between the two direct ISE methods include differences in bicarbonate and glucose concentrations between the blood sample measured in the blood gas POC apparatus and the serum sample measured in the apparatus of the main hospital laboratory, and a high level of blood hemoglobin resulting in a spurious decrease in the [Na]_S_ measured in whole blood with the direct ISE [151]. Finally, influences of hypernatremia and blood pH values on the measurement of [Na]_S_ by different ISE technologies have been reported [152].

### 5.8. Clinical Conditions Associated with Elevated Serum Solids Content

Using an indirect ISE will report a spuriously low sodium value on every serum sample with a high SSC. Pseudohyponatremia, whether it has been reported or not, has the same frequency as high SSC values in these conditions. A list of such conditions is provided in Table 4, which was composed from material contained in the reviews by Liamis and co-authors [153], and Koumpis and collaborators [154]. The Supplementary Material Section provides further information about these conditions.

## 6. Frequency of Spurious Serum Sodium Measurements

Table 3 and Table 4 show conditions in which pseudohyponatremia is probably frequent. A small number of reports studied the frequencies of pseudohyponatremia, pseudonormonatremia and pseudohypernatremia. Overlack and co-authors reported a frequency of 46.7% for asymptomatic low [Na]_S_ values, and a highly significant statistical association between low [Na]_S_ values and high blood viscosity values in 15 patients with multiple myeloma [41]. Lang and co-investigators reported equal frequencies of (1.3%) for hyperproteinemia and hypoproteinemia (serum proteins > 80 g/dL and <50 g/L respectively) in serum samples that were submitted for the measurement of urea and electrolytes. In both the hyperproteinemic and hypoproteinemic samples, the [Na]_S_ values were measured using both indirect and direct ISE approaches. In the hyperpoteinemic samples, the frequency of clinically significant pseudohyponatremia, defined as a [Na]_S_ value by direct ISE exceeding the value by indirect ISE by ≥4 mmol/L, was 16.1%. In the hypoproteinemic samples, the frequencies of both pseudonormonatremia and pseudohypernatremia were 1%. 

Chow and co-investigators reported an 85% frequency of hypoproteinemia in the sera from critically ill patients. In these sera, the [Na]_S_ values from direct ISE (140 ± 5.0 mmol/L) were significantly higher than the corresponding values via indirect ISE (136.5 ± 5.2 mmol/L), while pseudonormonatremia was noted in 19% and pseudohypernatremia in 8% of the serum samples [155]. Lava and co-authors estimated that [Na]_S_ values measured via direct ISE exceeded by ≥4 mmol/L the corresponding values measured via indirect ISE in 25% of the serum samples obtained from critically ill patients [156]. Katrangi and coinvestigators reported an inversely proportional difference between [Na]_S_ values measured with indirect and direct ISE, with 69% of the samples differing by ≥4.0 mmol/L [157]. Liamis and co-authors reported that 27.3% of the low [Na]_S_ values obtained on hospital admission for various alcohol-related conditions were cases of pseudohyponatremia. Their diagnosis of pseudohyponatremia was based on normal serum osmolality, severe hypertriglyceridemia, and increased [Na]_S_ values as the plasma levels of triglycerides decreased [158]. In 98 plasma samples collected from critically ill patients, Langelaan and collaborators reported that one of the six samples (16.7%) in which hyponatremia was reported via indirect ISE measurement was shown to be pseudohyponatremia with the direct ISE measurement [159].

## 7. Management of Pseudohyponatremia—Future Developments

Encountering pseudohyponatremia is inevitable, because most clinical laboratories still measure the [Na]_S_ using the indirect ISE method. The risk of iatrogenic complications accompanies pseudohyponatremia if this is not diagnosed promptly and is mismanaged. Both restriction of fluid intake [98] and saline infusion [19,92,108,160] have been inadvertently used to treat misdiagnosed pseudohyponatremia. Severe neurological manifestations [19] and deaths [91,160] have been reported following hypertonic saline infusion, leading to a rapid rise in the [Na]_SW_ to extreme levels in patients with pseudohyponatremia. The magnitude of the spurious measurement and the subsequent risk of inappropriate treatment increase in parallel with the magnitude of the difference in serum solids from normal values (Table 1 and Table 2).

Clinicians should be aware of their laboratory’s method for measuring the [Na]_S_. If the laboratory uses a direct ISE, then a clinician can accept the [Na]_S_ result at face value. However, if the laboratory uses an indirect ISE, then a [Na]_S_ lower than 137 mmol/L requires further examination to determine whether this value represents true hyponatremia or pseudohyponatremia. When an indirect ISE or FES is used, the levels of serum proteins and lipids should be measured, along with the [Na]_S_, in order to calculate the SSC from one of the available formulas, and the [Na]_SW_ [12]. The method for measuring the [Na]_S_ has not been addressed properly in the literature. Only 17% of the published studies in hyponatremia that were analyzed in the systematic review by Malandrini and coinvestigators provided information about the method for measuring the [Na]_S_ [25]. The recognition of simple pseudohyponatremia should change the focus of the management from hyponatremia to the condition which caused the high SSC. The management of pseudohyponatremia combined with other dysnatremias should address both the condition causing the pseudohyponatremia, and the condition causing the additional dysnatremias.

Future developments in the prevention of pseudohyponatremia must address the reality that the [Na]_SW_ and not the [Na]_S_ represents the important parameter that determines the biological functions of sodium. It was shown earlier that (a) the indirect ISE approach reports [Na]_S_ values that are close to the true values at all SWC values, but the [Na]_SW_ values that are computed using these [Na]_S_ values are accurate only when the SWC is 0.93 (Table 1 and Table 2); and (b) the direct ISE method reports [Na]_S_ values which indirectly indicate the true [Na]_SW_ values throughout the range of the SWC values, but these [Na]_S_ values are computed assuming an SWC of 0.93 only; therefore, they are only accurate at an SWC of 0.93. These findings suggest the following two measures:

The obvious first action to prevent pseudohyponatremia consists of using direct ISE devices for all measurements of the sodium concentration. Using this measure, pseudohyponatremia will be eliminated, with rare exceptions. The second action that should be pursued following the use of direct ISEs consists of changing the reported sodium concentration from the [Na]_S_ to the [Na]_SW_ [12]. This will require a recalibration of the direct ISE’s instruments, changing the normal range of sodium concentration, and reevaluating the target values of correcting the [Na]_SW_ in hyponatremia. The calculation of the volume of non-isotonic saline that is infused to change the [Na]_SW_ by a specific value, computed by any of the published formulas [161,162], requires accounting for differences between the sodium concentration in serum water and in the interstitial fluid compartments. The proteins in serum are polyanions that attract sodium, while interstitial fluids have low protein concentrations and a lower sodium concentration in their water compartment than in serum water. The differences in sodium concentration between serum water and water in interstitial fluids are quantified using the equations expressing the Gibbs–Donnan equilibrium [163].

Introducing new methods for measuring the [Na]_SW_ into clinical practice constitutes another potential future development. Two methods, field mass spectrometry and enzymatic determination, are worth exploring: field mass spectrometry is a promising recent technology for measuring electrolyte concentrations in biological fluids [164,165]; its measurement of the sodium concentration based on specific enzyme (β-galactosidase) activation by sodium ions has been applied in certain clinical conditions, e.g., isolation laboratories for emerging infectious diseases [26,166,167]. The Supplementary Material Section S1 provides further information about conditions causing hyperlipidemia [153,154,168,169,170,171,172,173,174,175,176,177,178,179,180,181,182,183,184,185,186,187,188,189,190,191,192,193,194,195,196,197].

## 8. Conclusions and Future Directions

Measurement of the [Na]_S_ with laboratory methods that require dilution of the serum sample carries the risk of pseudohyponatremia when the SSC is higher than the normal value of 0.07 (7% of serum volume), with adverse outcomes if a spuriously low [Na]_S_ value is treated. The possibility of pseudohyponatremia should be investigated when low [Na]_S_ values are reported via a method that requires pre-measurement dilution of the serum samples from patients with clinical conditions that cause increases in the SSC (hyperproteinemia, hyperlipidemia). Measurement of the [Na]_S_ with methods that do not require serum dilution will eliminate almost all cases of pseudohyponatremia.

## Figures and Tables

**Table 1:** Electrolyte exclusion effect. Actual [Na]_S_ values in sera with three different solid contents and the same [Na]_SW_ (151 mmol/L).

Serum Solids Content	Serum Water Content	[Na]_S_ (mmol/L)
0.07	0.93	0.93 × 151 = 140.4
0.14	0.86	0.86 × 151 = 129.9
0.21	0.79	0.79 × 151 = 119.3

[Na]_S_ = sodium concentration in serum; [Na]_SW_ = sodium concentration in serum water.

**Table 2:** Dilution effect. Measured [Na]_S_ after 1:31 (serum volume: diluent plus serum volume) pre-measurement dilution in sera with three different solid contents and the same [Na]_SW_ (151 mmol/L).

Component	SSC = 0.07 SWC = 0.93	SSC = 0.14 SWC = 0.86	SSC = 0.21 SWC = 0.79
Diluent, L	0.3	0.3	0.3
Serum, L	0.01	0.01	0.01
Serum sample water, L	0.0093	0.0086	0.0079
Total sample water, L	0.3093 (0.3 + 0.0093)	0.3086 (0.3 + 0.0086)	0.3079 (0.3 + 0.0079)
Dilution factor serum water (38)	33.2581 (0.3093/0.0093)	35.8837 (0.3086/0.0086)	38.9747 (0.3079/0.0079)
Sodium content of sample, mmoL	1.4043 (0.0093 × 151)	1.2986 (0.0086 × 151)	1.1929 (0.0079 × 151)
[Na]_DSW_, mmol/L	4.540 (1.4043/0.3093)	4.2080 (1.2986/0.3086)	3.8743 (1.1929/0.3079)
[Na]_SW_^1^, mmol/L	151.0 (4.540 × 33.2581)	140.0 (4.2080 × 33.2581)	128.9 (3.8743 × 33.2581)
[Na]_S_^1^, mmol/L	140.4 (151 × 0.93)	130.2 (140 × 0.93)	119.8 (128.9 × 0.93)

SSC = plasma solid content; SWC = plasma water content; [Na]_DSW_ = sodium concentration in the water of the diluted sample (the measured value); [Na]_SW_ = sodium concentration in serum water calculated by combining [Na]_DSW_ by the dilution factor for serum water; [Na]_S_ = sodium concentration in serum calculated by multiplying [Na]_SW_ by SWC; ^1^ all calculations of [Na]_SW_ and [Na]_S_ were carried out assuming an SWC of 0.93 (dilution factor of serum water of 33.2581).

**Table 3:** Reported cases of pseudohyponatremia.

High Serum Solids Component	Clinical Condition	References
Hyperproteinemia	Multiple myeloma	[39,62,63,64,65,66,67,68,69]
	Monoclonal gammopathies	[70]
	Waldenström’s macroglobulinemia	[71]
	HIV disease (hypergammaglobulinemia)	[72,73]
	Immunoglobulin infusion	[40,74,75,76]
Hypertriglyceridemia	Pancreatitis	[18,77,78,79,80]
	Acute or chronic alcoholism	[64]
	Asparaginase treatment	[81,82,83,84]
	Diabetic ketoacidosis	[85,86,87,88,89,90,91]
	Type 2 diabetes poorly controlled	[92]
	Genetic defects (lipoprotein lipase)	[93]
	Lipoproteinemia, types I and V	[31]
Hypercholesterolemia	Obstructive/cholestatic jaundice	[94,95,96]
	Pancreatic cancer with biliary obstruction	[97,98]
	Primary biliary cirrhosis	[41,99,100,101]
	Drug-induced cholestatic hepatitis	[102,103]
	Graft-versus-host liver disease	[104,105,106,107,108]
	Hepatitis	[109]
	Genetic defects (Alagille syndrome)	[110]

**Table 4:** Clinical conditions causing increased serum solids.

High Serum Solids Component	Clinical Condition
Hypergammaglobulinemia	Cirrhosis, Autoimmune hepatitis
	Alcoholic liver disease, Hepatitis C
	Interferon infusion
	POEMS syndrome
	Castleman’s disease
	Post-transplant monoclonal gammopathies
	Chronic lymphocytic leukemia
	Cryoglobulinemia, Cold agglutinin disease
	Gaucher’s disease
Hypertriglyceridemia	Alcoholism
	Interferon infusion
	Diabetes mellitus, Obesity
	All-trans-retinoic acid (ATRA)
Hypercholesterolemia	Diabetes mellitus
	Stem cell transplantation
	Non-Hodgkin’s lymphoma
Mixed hyperlipidemia	Diabetes mellitus
	Nephrotic syndrome from various causes
	Hemophagocytic lymphohistiocytosis
	Intravenous lipid emulsions
	Parenteral nutrition in COVID-19

POEMS = polyneuropathy, organomegaly, endocrinopathy, monoclonal protein, skin changes; COVID-19 = coronavirus disease of 2019.

## Data Availability

No new data were created or analyzed in this study. Data sharing is not applicable to this article.

## Supplementary Materials

The following supporting information can be downloaded at: https://www.mdpi.com/article/10.3390/jcm12124076/s1, Section S1-Clinical conditions causing increased serum solid content [168,169,170,171,172,173,174,175,176,177,178,179,180,181,182,183,184,185,186,187,188,189,190,191,192,193,194,195,196,197].
