# Supplementary material for: Pseudohyponatremia: Mechanism, Diagnosis, Clinical Associations and Management
Source: J Clin Med. 2023 Jun 15;12(12):4076. doi: 10.3390/jcm12124076 (PMC10299669; doi:10.3390/jcm12124076)
Supplement: Supplementary file 1 [file jcm-12-04076-s001.zip › jcm-2436122-supplementary.pdf]

Supplemental Material

Title: Pseudohyponatremia: Mechanism, Diagnosis, Clinical Associations and Management

Authors: Fahad Aziz 1, Ramin Sam 2, Susie Q. Lew 3, Larry Massie 4, Madhukar Misra 5, Maria-Eleni Roumelioti 6, Christos P. Argyropoulos 6, Todd S. Ing 7 and Antonios H. Tzamaloukas 8

Corresponding author: Christos P. Argyropoulos, Department of Medicine, Division of Nephrology, University of New Mexico School of Medicine, Albuquerque, NM 87106, USA

Section S1-Clinical conditions causing increased serum solid content

Conditions causing hyperproteinemia

Hypergammaglobulinemia represents a major laboratory feature of various forms of hepatitis or cirrhosis [153]. High SSC is such patients may be caused by both hyperproteinemia and hypercholesterolemia from biliary obstruction as noted earlier. Interferon administration represents another source of hyperproteinemia [153–154]. Hyperproteinemia is caused by elevated levels of light chains, almost always of the lambda type, in POEMS (polyneuropathy, organomegaly, endocrinopathy, monoclonal protein, skin changes) syndrome [168] and by high blood levels of inflammatory cytokines in Castleman's disease [169]. Hypergammaglobulinemia is encountered in 25%-91% of the cases and monoclonal gammopathy in 1%-35% of the cases of Gaucher's disease, which is an autosomal recessive lysosomal storage disease caused by mutations of the 1q21 GBA1 gene located in chromosome 1 [170].

Conditions causing hypertriglyceridemia

Excessive ethanol consumption leads to hypertriglyceridemia through increased secretion of VLDL, impaired lipolysis and increased fluxes to the liver of fatty acids from adipose tissue. Obesity increases the risk of severe hypertriglyceridemia secondary to ethanol consumption [171]. Alpha-interferon and other cytokines stimulate lipid synthesis in the liver [172]. Administration of alpha-interferon for treatment of hepatitis C led repeatedly to transient, but pronounced, increases in serum triglycerides [173]. Several studies document hyperlipidemias in diabetic patients [174-183]. Type 2 and poorly controlled type 1 diabetes mellitus and obesity lead to increased peripheral lipolysis, increased delivery of fatty acids to the liver and hypertriglyceridemia [174]. Juvenile diabetics may have combined hypercholesterolemia and hypertriglyceridemia [181]. Type 2 diabetics have frequent hyperlipidemias, including types II, IV, and combined types II and IV [183]. Hypertriglyceridemia constitutes the dose-limiting toxicity of the antineoplastic agent all-trans-retinoic acid (ATRA) [184].

Conditions causing hypercholesterolemia and/or combined hyperlipidemia

Cholestasis with elevated serum levels of lipoprotein X has caused pronounced hypercholesterolemia in graft-versus-host disease with liver involvement [185] and non-Hodgkin's lymphoma [186]. Nephrotic syndrome secondary to various types of glomerular disease causes changes in multiple proteins involved in the metabolism of lipids [187] resulting

in severe combined hyperlipidemia and hypercholesterolemia [187–190]. In this case, spuriously low [Na]s measurements will be reported by indirect ISE only in cases in which the increase in SSC caused by hyperlipidemia is larger than the decrease secondary to low serum protein values.

In hemophagocytic lymphohistiocytosis, a syndrome characterized by abnormal activation of T-cells and overproduction of inflammatory cytokines, hypertriglyceridemia and hypercholesterolemia are frequent laboratory findings [191–192]. Various preparations of infused lipid emulsions result directly in hyperlipidemia, but also increase the risk of hepatic damage [193–194] including cholestasis [195]. The risk of hypertriglyceridemia in COVID-19 patients receiving parenteral nutrition was increased by simultaneous administration of lopinavir/ritonavir or propofol and the presence of obesity [196]. Koch and coauthors evaluated in vitro pseudohyponatremia in serum samples containing lipid preparations and advocated the use of direct ISE for measuring [Na]s at lipemia indices above a cut-off value of 700 [197].

References

153. Liamis, G.; Filippatos, T.D.; Liontos, A.; Elisaf, M.S. Hyponatremia in patients with liver diseases: not just a cirrhosis-induced hemodynamic compromise. *Hepatol Int*. **2016**, *10*, 762-772.
154. Koumpis, E.; Florentin, M.; Hatzimichael, E.; Liamis, G. Hyponatremia in patients with hematologic diseases. *J Clin Med*. **2020**, *9*, 3721.
168. Dispenzieri A. POEMS syndrome: 2019 update on diagnosis, risk-stratification, and management. *Am J Hematol*. **2019**, *94*, 812-827.
169. Liu, A.Y.; Nabel, C.S.; Finkelman, B.S.; Ruth, J.; Kurzrock, R.; van Rhee, F.; Krymskaya, V.P.; Kelleher, D.; Rubenstein, A.H.; Fajgenbaum, D.C. Idiopathic multicentric Castleman's disease: a systematic literature review. *Lancet Haematol*. **2016**, *3*, e163-e175.
170. Stirneman, J.; Belmatoug, N.; Camou, F.; Serratrice, C.; Froissart, R.; Caillaud, C.; Levade, T.; Astudillo, L.; Serratrice, J.; Brassier, A.; Rose, C.; Billette de Villemeur, T.; Berger M.G. A review of Gaucher disease pathophysiology, clinical presentation and treatments. *Int J Mol Sci*. **2017**, *18*, 441.

171. Klop, B.; do Rego, A.T.; Cabezas, M.C. Alcohol and plasma triglycerides. *Curr Opin Lipidol*. **2013**, *24*, 321-326.
172. Feingold, K.R.; Soued, M.; Serio, M.K.; Moser, A.H.; Dinarello, C.A.; Grunfeld. C. Multiple cytokines stimulate hepatic lipid synthesis *in vivo*. *Endocrinology*. **1989**, *125*, 267-274.
173. Graessle, D.; Bonacini, M.; Chen, S. Alpha-inteferon and reversible hypertriglyceridemia. *Ann Intern Med*. **1993**, *118*, 316-317.
174. Harris, L.V.D., Albrink, M.J.; Van Eck, W.F.; Man, E.B.; Peters, J.P. Serum lipids in diabetic acidosis. *Metabolism*. **1953**, *2*, 120-132.

175. Hamwi G.J.; Garcia, O.; Kruger, F.A.; Gwinup, G.; Cornwell, D.G. (1962) Hyperlipidemia in uncontrolled diabetes. *Metabolism*. **1962**, *11*, 850-862.
176. Sterky, G.; Larsson, Y.; Persson, B. Blood lipids in diabetic and non-diabetic schoolchildren. *Acta Paediatr (Stockh)*. **1963**, *52*, 11-21.
177. Bagdade, J.D.; Porte, D. Jr.; Bierman, E.L. Diabetic lipidemia. A form of acquired fat-induced lipemia. *N Engl J Med*. **1967**, *276*, 427-433.
178. Chance, G.W.; Albutt, E.C.; Edkins, S.M. Serum lipids and lipoproteins in untreated diabetic children. *Lancet*. **1969**, *1(7606)*, 1126-1128.
179. Wilson, D.E.; Schreibman, P.H.; Day, V.C.; Arky, R.A. Hyperlipidemia in an adult diabetic population. *J Chron Dis*. **1970**, *23*, 501-506.

180. Hayes, T.M. Plasma lipoproteins in adult diabetes. *Clin Endocrinol (Oxf)*. **1972,** 1, 247-251.
181. Billimoria, J.D.; Isaacs, A.J.; Melki, K. A lipid and liporotein profile of treated and untreated diabetics. *Ann Clin Biochem*. **1976**, *13*, 315-321.
182. Chace, H.P.; Glasgow, A.M. Juvenile diabetes mellitus and serum lipids and lipoprotein levels. *Am J Dis Child*. **1976**, *130*, 1113-1117.
183. Subramanian, S.; Chait, A. Hypertriglyceridemia secondary to obesity and diabetes. *Biochim Biophys Acta*. **2012**, *1821*, 819-825.
184. Conley, B.A.; Egorin, M.J.; Sridhara, R.; Finley, R.; Hemady, R.; Wu, S.; Tait, N.S.; Van Echo, D.A. Phase I clinical trial of all-trans-retinoic acid with correlation of its pharmacokinetics and pharmacodynamics. *Cancer Chemother Pharmacol*. **1997**, *39*, 291-299.
185. Joukhadar, R.; Chiu, K. Severe hypercholesterolemia in patients with graft-vs-host disease affecting the liver after stem cell transplantation. *Endocr Pract*. **2012**, *18*, 90-97.
186. Chudy-Onwugaje, K.; Anyadike, N.; Tsirlin, Y.; Mayer, I.; Rahmani, R. Severe hypercholesterolemia: a unique presentation of non-Hodgkin's lymphoma in a patient with neurofibromatosis type 1. *Case Rep Gastrointest Med*. **2014**, *2014*, 579352.
187. Vaziri, N.D. Disorders of lipid metabolism in nephrotic syndrome: mechanisms and consequences. *Kidney Int*. **2016**, *90*, 41-52.

188. Joven, J.; Villabona, C.; Vilella, E.; Masana, L.; Albertí, R.; Vallés, M. Abnormalities of lipoprotein metabolism in patients with the nephrotic syndrome. *N Engl J Med*. **1990**, *323*, 579-584.

189. Wheeler, D.C.; Bernard, D.B. Lipid abnormalities in the nephrotic syndrome: causes, consequences, and treatment. *Am J Kidney Dis*. **1994**, *23*, 331-346.

190. Sar, F.; Taylan, I.; Kutlu, C.; Cayma, M.S.; Tatli, E.; Kazancioglu, R. Amyloidosis in a patient with autosomal dominant polycystic kidney disease and tuberculosis: a case report. *Int Urol Nephrol*. **2007**, *39*, 655-659.

191. Jordan, M.B.; Allen, C.E.; Weitzman, S.; Filipovich, A.H.; McLain, K.L. How I treat hemophagocytic lymphohistiocytosis. *Blood*. **2011**, *118*, 4041-4052.

192. Li, J.; Wang, Q.; Zheng, W.; Ma, J.; Zhang, W.; Wang, W.; Tian, X. Hemophagocytic lymphohistiocytosis: clinical analysis of 103 adult patients. *Medicine (Baltimore)*. **2014**, *93*, 100-105.

193. Hojsak, I.; Kolaček, S. Fat overload syndrome after the rapid infusion of SMOFlipid emulsion. *JPEN J Parent Enteral Nutr*. **2014**, *38*, 119-121.
194. Waitzberg, D.L.; Torrinhas, R.S. The complexity of prescribing intravenous lipid emulsions. *World Rev Nutr Diet*. **2015**, *112*, 150-162.

195. Li, X.X.; Cheng, Y.C.; Zhai, S.D.; Yao, P.; Zhan, S.Y.; Shi, L.W. Risk of liver injury associated with intravenous lipid emulsions: a prescription sequence symmetry analysis. *Front Pharmacol*. **2021**, *12*, 589091.
196. Villa López, G.; Valero Zanuy, M.A.; González Barrios, I.; Maíz Jiménez, M.; Gomis Muñóz, P.; León Sanz, M. Acute hypertriglyceridemia in patients with COVID-19 receiving parenteral nutrition. *Nutrients*. **2021**, *13*, 2287.
197. Koch, C.D.; Vera, M.A.; Messina, J.; Price, N.; Durant, T.J.S., El-Khoury, J.M.

Preventing pseudohyponatremia: Intralipid^{R}-based lipemia cutoffs for sodium are inappropriate.
Clin Chim Acta. **2021**, *520*, 63-66.
